# CRISPR-Cas9 Gene Therapy: Non-Viral Delivery and Stimuli-Responsive Nanoformulations

**DOI:** 10.3390/molecules30030542

**Published:** 2025-01-24

**Authors:** Hyunwoo Lee, Won-Yeop Rho, Yoon-Hee Kim, Hyejin Chang, Bong-Hyun Jun

**Affiliations:** 1 Department of Bioscience and Biotechnology, Konkuk University, Seoul 05029, Republic of Korea; skywalker0202@konkuk.ac.kr (H.L.); yoonhees@konkuk.ac.kr (Y.-H.K.); 2 School of International Engineering and Science, Jeonbuk National University, Jeonju 54896, Republic of Korea; rho7272@jbnu.ac.kr; 3 Division of Science Education, Kangwon National University, 1 Gangwondaehakgil, Chuncheon-si 24341, Republic of Korea

**Keywords:** CRISPR-Cas9, gene therapy, non-viral delivery systems, stimuli-responsive, stimuli-responsive nanomaterials

## Abstract

The CRISPR-Cas9 technology, one of the groundbreaking genome editing methods for addressing genetic disorders, has emerged as a powerful, precise, and efficient tool. However, its clinical translation remains hindered by challenges in delivery efficiency and targeting specificity. This review provides a comprehensive analysis of the structural features, advantages, and potential applications of various non-viral and stimuli-responsive systems, examining recent progress to emphasize the potential to address these limitations and advance CRISPR-Cas9 therapeutics. We describe how recent reports emphasize that nonviral vectors, including lipid-based nanoparticles, extracellular vesicles, polymeric nanoparticles, gold nanoparticles, and mesoporous silica nanoparticles, can offer diverse advantages to enhance stability, cellular uptake, and biocompatibility, based on their structures and physio-chemical stability. We also summarize recent progress on stimuli-responsive nanoformulations, a type of non-viral vector, to introduce precision and control in CRISPR-Cas9 delivery. Stimuli-responsive nanoformulations are designed to respond to pH, redox states, and external triggers, facilitate controlled and targeted delivery, and minimize off-target effects. The insights in our review suggest future challenges for clinical applications of gene therapy technologies and highlight the potential of delivery systems to enhance CRISPR-Cas9’s clinical efficacy, positioning them as pivotal tools for future gene-editing therapies.

## 1. Introduction

Gene therapies primarily involve the direct manipulation of DNA or RNA to treat or prevent diseases [1]. The strategies for gene therapy are diverse, enabling not only the correction, replacement, or removal of genetic mutations but also the creation of inactive mutations in pathogen genomes to combat infectious diseases or induce somatic mutations for therapeutic and protective purposes [2]. Gene therapy is now considered to be applicable to treat various genetic disorders, including blood diseases, cancer, AIDS, diabetes, cardiovascular diseases, and neurodegenerative disorders [3], and to date, more than 2000 gene therapy clinical trials have been conducted worldwide [4]. Among the various gene therapies, including somatic cell gene therapy, germline gene therapy, and gene therapy vaccines, CRISPR-Cas9 technology, which modifies DNA through gene editing, has attracted much attention due to its potential to revolutionize the approach to genetic diseases and other challenging health conditions [5].

CRISPR-Cas9 is currently the most widely used tool for gene editing in the scientific community, enabling precise targeting, and followed modifications such as insertions, deletions, and base substitutions for specific genetic loci [6,7]. CRISPR-Cas9 even offers potential for effectively correcting numerous genetic diseases, including those resulting from single-gene mutations [8]. The first clinical trial utilizing CRISPR-Cas9 was approved in 2016, where the treatment allowed reactivation of T cells against lung cancer by editing patient blood cells to remove PD-1 [9]. Since then, dozens of clinical trials involving CRISPR-Cas9 gene editing have been approved by the National Institutes of Health (NIH), expanding to the treatments for lymphomas, HIV, and genetic disorders, highlighting its potential and significance in gene therapy [10].

Although CRISPR-Cas9-based gene therapies exhibit significant potential for treating various genetic diseases [11], their widespread clinical application remains limited [12]. To maximize the efficacy of gene therapy, the CRISPR-Cas9 system must be delivered safely and efficiently to target cells [13]. However, the direct administration of the CRISPR-Cas9 system into the bloodstream can trigger immune responses and pose the risk of damaging healthy cells, leading to reduced drug efficacy and severe side effects [14]. Particularly, Cas9 and gRNA, which are foreign elements, are unstable in vivo and pose challenges to tissue-specific targeting [15]. The Cas9 protein within the ribonucleoprotein (RNP) complex is relatively large (160 kDa), and single guide RNA (sgRNA) carries a negative charge, resulting in low cellular uptake efficiency owing to electrostatic repulsion with cell membranes [16]. These physical barriers present significant challenges to achieving high-precision gene editing, particularly in areas such as cancer therapy, where treatment efficacy may fall short of expectations [17].

To address these issues, efficient strategies for delivery of CRISPR-Cas9 are required for expanding the impact of this technology. CRISPR-Cas9 components should be delivered through delivery systems designed to ensure targeted delivery to tissues, rather than direct administration into the bloodstream [18]. The delivery systems should be able to facilitate the safe entry of CRISPR-Cas9 components into cells, ensure system stability, and minimize immune responses [19]. Various delivery systems have been developed to deliver CRISPR-Cas9 to target cells and can be broadly categorized into viral and non-viral delivery systems [20].

Traditional viral delivery systems deliver the CRISPR-Cas9 system into cells, offering the advantage of easily transferring genetic information to cells and providing high gene-editing efficiency [21]. However, they can elicit immune responses in the body and may lead to persistent off-target effects owing to sustained gene-editing activity within cells [22]. Additionally, the limited packaging capacity of viral vectors restricts their application [23]. To overcome these limitations, recent studies have focused on non-viral delivery systems [24]. Advances in nanotechnology have highlighted non-viral systems because of their low immunogenicity, physicochemical properties that can be tuned through surface functionalization, and enhanced stability in packaging large molecules [25]. However, the most significant challenge associated with non-viral CRISPR-Cas9 delivery systems is their markedly lower efficiency compared to viral delivery systems [26]. For non-viral delivery systems to function effectively, several biological barriers must be overcome. For instance, they must evade recognition and clearance by immune cells, withstand enzymatic degradation by nucleases and proteases, and, after successful internalization into the target cells, escape from endosomes to release CRISPR-Cas9 into the cytoplasm for subsequent nuclear delivery [27].

To address these challenges, non-viral delivery systems can be designed to possess stimulus-responsive properties, enabling the activation of their payloads in response to specific physiological environments or external stimuli [28]. Such stimulus-responsive nanoformulations facilitate spatial control by minimizing off-target editing in non-target organs or tissues while enabling tissue-specific genome editing [29]. Furthermore, temporal control can mitigate off-target effects and immunogenicity arising from prolonged CRISPR-Cas9 expression and activity, supporting transient and regulated genome editing. These features ultimately enhance the safety and efficiency of gene therapy [30].

**Figure 1:**
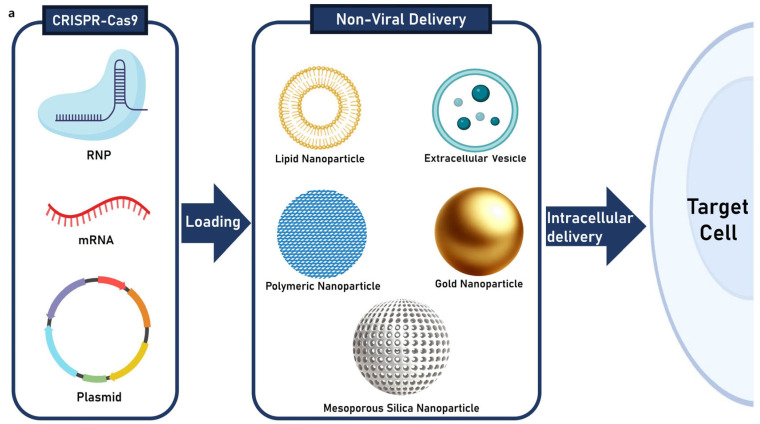

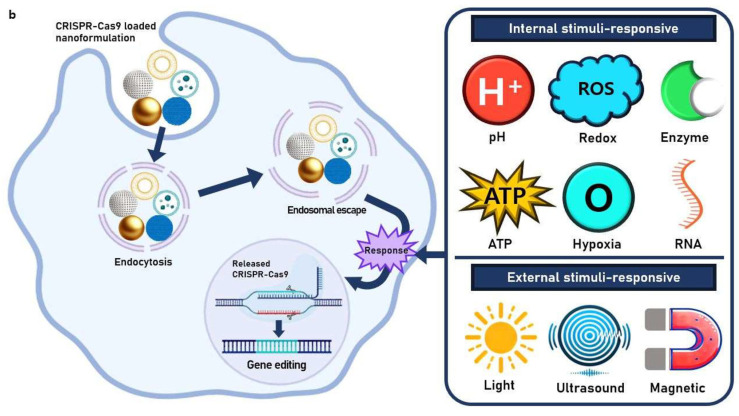
Overview of non-viral systems for CRISPR-Cas9 delivery and gene editing. (**a**) Non-viral delivery systems for loading and targeted intracellular delivery of CRISPR-Cas9 systems. (**b**) Mechanism of stimuli-responsive CRISPR-Cas9 nanoformulations for enhanced gene editing.

In summary, the CRISPR-Cas9 gene-editing system requires the simultaneous delivery of two key components: Cas9 and sgRNA. For in vivo delivery, these components are loaded onto a non-viral delivery platform. Once loaded, they are injected into the body and subsequently achieve intracellular delivery to the target cells. Additionally, the CRISPR-Cas9-loaded nanoplatform delivered to target cells is primarily internalized into the cells via endocytosis. Under specific conditions, stimuli-responsive CRISPR-Cas9 nanoplatforms can facilitate endosomal escape or, after escape, help the CRISPR-Cas9 gene-editing system migrate into the nucleus to perform gene editing (Figure 1).

Understanding each delivery system is crucial for the broad clinical application of CRISPR-Cas9-based gene therapeutics. However, no comprehensive review has been published that summarizes the recent research trends and integrates a unified perspective on the CRISPR-Cas9 mechanism, non-viral delivery systems, and stimuli-responsive nanostructured formulations. This review presents the biomolecular methods used in CRISPR-Cas9 delivery and explains the mechanisms underlying gene editing. It focuses on non-viral delivery systems for CRISPR-Cas9, detailing the structural characteristics, advantages, and potential future directions of various nanoparticles. Furthermore, recent advancements in stimuli-responsive nanostructured formulations designed for the controlled delivery of CRISPR-Cas9 systems have summarized their applicability in gene therapeutics. This highlights an innovative strategy to enhance the stability of genome editing technologies while simultaneously expanding their scope of application through the unique characteristics and inherent functionalities of stimulus-responsive nanoformulations leveraging nanotechnology. In conclusion, this approach provides a comprehensive perspective that spans from the gene-editing mechanisms of CRISPR-Cas9 to the genomic engineering of delivery systems, offering a cohesive framework for the effective development of CRISPR-Cas9-based gene therapeutics.

## 2. CRISPR-Cas9 Gene Editing Mechanism

**Figure 2:**
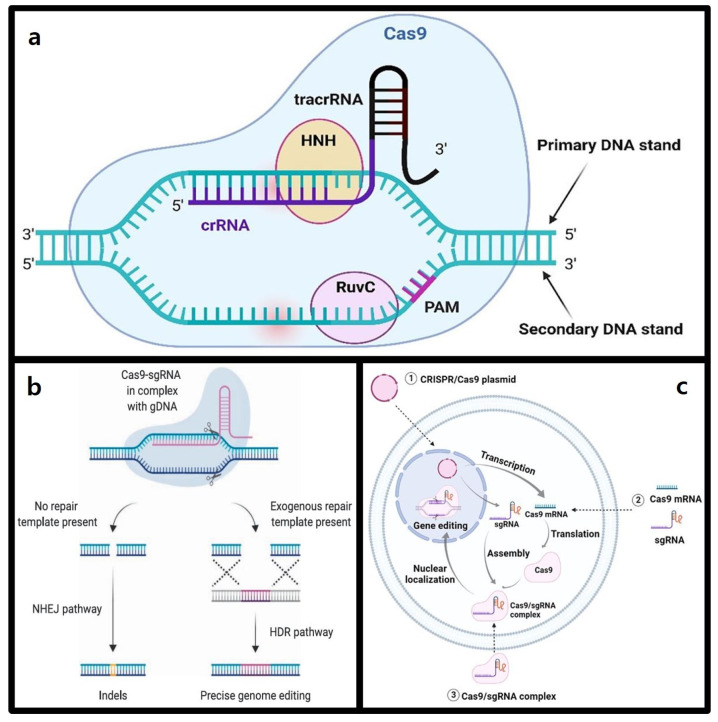
Comprehensive overview of the structural mechanism and gene editing pathways of the CRISPR-Cas9 system. (**a**) Binding and DNA cleavage mechanism of Cas9 protein and gRNA [60]. (**b**) CRISPR-Cas9 mediated gene editing and repair pathways [61]. (**c**) Mechanism of intracellular delivery and activation of CRISPR-Cas9 [62].

The CRISPR-Cas9 system is a gene-editing technology derived from the immune systems of bacteria and archaea and is based on a natural immune defense mechanism that targets and cleaves foreign genetic material with a protospacer-adjacent motif (PAM) [31]. This system operates through two main components of gene editing as described in Figure 2a: a single guide RNA (sgRNA) and the Cas9 nuclease [32].

The sgRNA guides Cas9 to the target DNA sequence, facilitating DNA binding and enabling precise cleavage at the target site [33]. The sgRNA consist of two elements: CRISPR RNA (crRNA) and trans-activating CRISPR RNA (tracrRNA) [34]. In bacteria and archaea with CRISPR systems, DNA fragments from degraded foreign genomes are integrated into the spacer region of the CRISPR array [35]. Upon reinvasion by the same genome, the CRISPR array is transcribed into pre-crRNA, which pairs with tracrRNA—a sequence complementary to the repeat regions of the CRISPR array [36]. Following transcription, tracrRNA binds to the Cas9 protein and forms a double-stranded RNA through complementary pairing with pre-crRNA [37]. Pre-crRNA is first cleaved by RNase III, then further processed by Cas9 to produce mature crRNA [38]. This mature crRNA targets DNA sequences, and forms functional sgRNAs when combined with tracrRNA [39].

Cas9 operates as an RNA-guided endonuclease, recognizing an adjacent PAM sequence in the target DNA to achieve precise double-strand breaks (DSBs) [40]. The PAM, a short conserved sequence in the invading DNA, is crucial for target DNA recognition and cleavage [41]. Cas9 contains two nuclease domains: HNH, which cleaves the DNA strand complementary to the guide RNA, and RuvC, which cleaves the non-target strand, resulting in double-strand breaks (DSBs) at specific sites [42].

The CRISPR-Cas9 system forms a Cas9-sgRNA ribonucleoprotein (RNP) complex, which identifies target DNA sequences based on PAM motifs (e.g., NGG) [43]. This complex facilitates the binding of the 20-nucleotide sequence at the 5’ end of the sgRNA to the target DNA, leading to Cas9-mediated DSBs [44].

These DSBs induced by CRISPR-Cas9 are repaired via two primary pathways as described in Figure 2b: non-homologous end joining (NHEJ) or homology-directed repair (HDR) [45]. NHEJ rapidly ligates broken DNA ends but may introduce insertions or deletions (indels), which can cause frameshift mutations and premature stop codons, effectively knocking out the targeted gene [46]. Conversely, HDR utilizes an exogenous donor template to achieve precise repair, enabling the insertion or modification of specific gene sequences and facilitating gene correction or knock-in [47]. Thus, CRISPR-Cas9 enables genome editing by cleaving target DNA, followed by gene disruption via NHEJ or precise sequence insertion through HDR [48]. This mechanism forms the foundation of CRISPR-Cas9’s transformative role in genome editing, positioning it as a revolutionary tool for gene therapy [49].

The CRISPR-Cas9 system requires the coordinated delivery of Cas9 and sgRNA, typically achieved through three main delivery methods as seen in Figure 2c: plasmid DNA, messenger RNA (mRNA), and RNP. Each has approach offers distinct advantages in terms of cellular function, speed, and efficiency [50].

Plasmid DNA is commonly used to deliver the genes encoding the CRISPR-Cas9 components (Cas9 protein and sgRNA) into target cells, serving as a stable and easily producible vector [51]. Once inside the cell nucleus, plasmid DNA undergoes transcription and translation to generate RNPs enabling sustained gene editing through long-term expression [52]. However, this method is limited by its dependence on the cell cycle, slow onset of expression, and low transfection efficiency due to the large size of plasmids [53].

In some applications, mRNA is used instead of plasmid DNA to bypass the limitations of transcription and translation, allowing for faster and more controlled genome editing [54]. However, mRNA is inherently unstable and prone to degradation by cytoplasmic RNase, resulting in transient expression [55].

The RNP delivery method has garnered significant attention in recent studies due to its simplicity and efficiency [56]. By bypassing transcription and translation steps, this approach enables immediate gene editing with the nucleus, achieving higher editing efficiency compared to DNA or mRNA delivery. In addition, the transient presence of RNP minimizes off-target effects, enhancing specificity [57]. However, the delivery of RNPs poses several challenges. The large molecular weight of the Cas9 protein, its requirement for sgRNA binding, and its inability to cross cell membranes hinder efficient loading and intracellular transport [58]. Furthermore, the purification of the highly active Cas9 protein is complex, and its size limits cellular entry, emphasizing the need for advanced nanodrug delivery systems capable of effectively transporting RNP cargo [59]. 

## 3. Non-Viral Delivery System of CRISPR-Cas9

The direct administration of CRISPR-Cas9-based gene therapeutics into the bloodstream can result in off-target effects, damaging heathy cells and causing potentially severe side effects [63]. Furthermore, rapid in vivo metabolism can significantly reduce therapeutic efficacy [64]. To address these challenges, non-viral delivery systems have been developed, with synthetic nanomaterials gaining considerable attention for their potential in drug delivery [65]. These systems must exhibit high biocompatibility, efficient delivery, and precise targeting capabilities [66]. Additionally, they should offer high loading capacity, controlled release, and inert properties to prevent adverse interactions with the gene-editing therapeutics they carry [67].

The selection of an appropriate delivery vehicle for CRISPR-Cas9 systems is essential to improving both effectiveness and efficiency of gene-editing therapies [68]. Recent advancements in nanotechnology have highlighted the potential of multifunctional nanocarriers in addressing these needs [69]. This chapter reviews the structural characteristics and recent developments in nonviral nanocarriers, focusing on lipid nanoparticles (LNPs), extracellular vesicles (EVs), polymeric nanoparticles, gold nanoparticles (AuNPs), and mesoporous silica nanoparticles (MSNs) shown in Figure 1a. Furthermore, it summarizes the unique advantages of each type of CRISPR-Cas9 system delivery, providing an overview of their applications and potential in advance gene-editing therapeutics.

### 3.1. Lipid-Based Nanoparticles

#### 3.1.1. Structure and Characteristics of Lipid Nanoparticles (LNPs)

LNPs are an advanced drug delivery platform designed to efficiently transport biomolecules, such as DNA and RNA, into cells. Structurally, LNPs are spherical vesicles composed of lipids with hydrophilic head groups and hydrophobic tail groups, which self-assemble into nanostructures in aqueous environments [70]. These nanostructures form due to the lipid’s physicochemical properties, which reduce interactions like electrostatic repulsion and steric hindrance, thereby enhancing LNP stability. Furthermore, the inherent biocompatibility and safety of LNPs allow them to evade the innate immune system and extend their circulation time [71]. This makes LNPs particularly effective for delivering nucleic acids and hydrophobic protein-based drugs with short circulation half-lives, enabling precise therapeutic gene editing with the CRISPR-Cas9 system.

The significance of LNPs, with their ability to preserve lipid physicochemical properties in aqueous environments, became apparent with the fact that many small molecule anticancer drugs have low aqueous solubility, requiring encapsulation strategies to enhance delivery [72]. Several liposome-based pharmaceutical formulations have since been approved, showcasing their versatility in encapsulating hydrophilic drugs within their aqueous core and hydrophobic drugs within their lipid bilayer [73]. However, liposome technologies face challenges such as complex manufacturing processes, scalability limitations, and restricted applications for small-molecule drugs.

**Figure 3:**
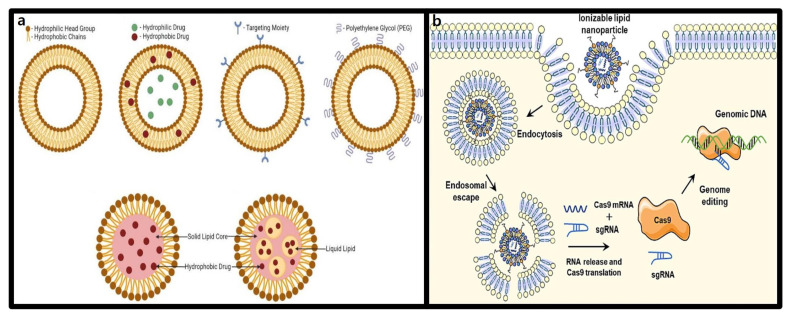
Structural characteristics of LNPs and CRISPR-Cas9 delivery mechanism. (**a**) Structural diversity of LNPs and surface functionalization for targeted delivery [75]. (**b**) LNP-based intracellular delivery and activation mechanism of the CRISPR-Cas9 system [78].

LNPs were developed to address these limitations. Similar to liposomes, LNPs are composed of phospholipids, cholesterol, and PEGylated lipids, but with the addition of ionizable cationic lipids. These cationic lipids facilitates electrostatic interactions with negatively charged nucleic acids, forming stable nanoparticles [74]. Unlike the bilayer structure of liposomes, LNPs can adopt diverse internal architectures influenced by their nucleic acid cargo, lipid composition, manufacturing methods, and processing conditions. Negatively charged biomolecules such as CRISPR-Cas9 plasmid DNA, mRNA, and gRNA can stably bind to LNPs via electrostatic interactions, enabling the formation of highly efficient delivery systems, as shown in Figure 3a. Due to these properties, LNPs have become the most widely used non-viral delivery system for nucleic acids, with extensive research into their therapeutic applications ongoing [75].

#### 3.1.2. Advantages of LNPs in CRISPR-Cas9 Delivery

The release of nucleic acids bound to cationic lipids following LNPs entry into cells is a critical step in effective nucleic acid delivery (Figure 3b). To improve delivery efficiency, extensive research has focused on the chemical optimization of cationic lipids in LNPs [76]. These cationic lipids contain ionizable amine structures with variable charges. At low pH, the amine groups acquire a positive charge, enabling binding to negatively charged cargo while maintaining a neutral state at physiological pH to reduce toxicity. Upon endosomal acidification during cellular uptake, the amine structures regain their positive charge, facilitating cargo release. Negatively charged lipids within the cell further promote nucleic acid release by neutralizing the charge of the cationic lipid carriers and disrupting electrostatic interactions. This mechanism allows LNPs to deliver nucleic acid drugs with highly stability and efficiency in vivo, enhancing intracellular delivery [77]. Beyond amine structures, the hydrophobic lipid tails and linkers in LNPs can also be chemically modified to design new lipid-based nanocarriers. Incorporating of biodegradable linkers, branched chains, and functional groups enhances the biocompatibility, efficacy, and targeting capabilities of lipid-based carriers [78].

The functionalization of LNPs has emerged as an effective strategy for delivering CRISPR-Cas9 therapeutics. Zhang et al. developed a polyethylene glycol-modified lipid nanocarrier (LPLNP) for delivering Cas9 plasmid/sgRNA to A375 cells. This system efficiently condensed and encapsulated the Cas9/sgPLK-1 plasmid into a core–shell structure (PLNP/DNA), achieving a transfection efficiency of 47.4% in A375 cells. In mouse models, intratumoral injection of the Cas9/sgPLK-1 plasmid into melanoma-bearing mice significantly downregulated polo-like kinase 1 (PLK-1) protein expression, resulting in over 67% inhibition of tumor growth [79].

A critical advantage of LNPs is their ability to target tissues with high precision. This capability is primarily achieved by modulating lipid composition, significantly reducing the off-target effects associated with non-specific delivery methods. LNPs demonstrate particularly high accumulation efficiency in the liver, making them suitable for treating liver diseases. The efficiency, scalability, and potential for single-administration treatments position LNP-based delivery systems as promoting candidates for clinical applications [80]. Intellia Therapeutics initiated the first clinical trial using LNPs as a delivery vector for CRISPR-Cas9, targeting hereditary transthyretin amyloidosis with polyneuropathy (ATTRv-PN) in NTLA-2001 therapy. This therapy aims to edit the TTR gene in hepatocytes. Interim clinical results published in 2021 showed that a high dose (0.3 mg/kg) of NTLA-2001 led to an average 87% reduction in serum TTR protein levels by day 28 post-treatment, while a low dose (0.1 mg/kg) achieved a 52% reduction. Mild adverse effects were observed, but they resolved quickly. A larger Phase 1 trial is currently in progress [81].

Despite these advances, challenges remain in targeting organs other than the liver, which is a key area for improvement. The development of tissue-specific LNP modification technologies is progressing rapidly through nanotechnology, expanding the applicability of gene-editing therapies [82]. These innovations are expected to drive the broader use of CRISPR-Cas9-based gene therapies, unlocking new possibilities in precision medicine and treatment of diverse diseases.

### 3.2. Extracellular Vesicles

#### 3.2.1. Structure and Characteristics of Extracellular Vesicles (EVs)

EVs are membrane-bound vesicles derived from endosomal compartments that are secreted into the extracellular environment following fusion with the plasma membrane. They play a crucial role in intercellular communication, transporting various biological molecules such as bioactive lipids, proteins, and nucleic acids, and are involved in the regulation of numerous physiological processes [83]. EVs are generally categorized into exosomes, microvesicles, and apoptotic bodies based on their size and formation mechanisms. However, overlapping size ranges, biomarkers, and compositions pose challenges to isolating pure EVs. For simplicity, this article collectively refers exosomes, microvesicles, and apoptotic bodies as EVs [84].

EVs can encapsulate biomacromolecules—including lipids, proteins, and nucleic acids—and deliver them to target cells via micropinocytosis, endocytosis, or direct membrane fusion, indicating the significant potential of EVs as delivery vehicles for the CRISPR-Cas9 system [85]. Moreover, EVs exhibit intrinsic targeting capabilities due to the sugars, proteins, and lipids on their surfaces, allowing efficient travel to cells similar to their origin. This targeting ability arises from shared surface receptors and matrix-binding proteins between EVs and their target cells [86].

However, two major challenges hinder the in vivo application of EVs: unstable targeting capability, which can result in off-target effects, and rapid clearance by immune cells. Addressing these challenges is crucial for improving the efficiency of EV-based CRISPR-Cas9 therapeutics, and active research is underway [87].

**Figure 4:**
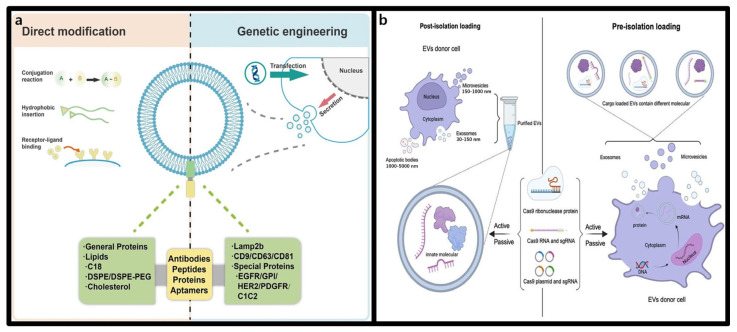
EVs engineering strategies for CRISPR-Cas9 Delivery. (**a**) Direct modification and genetic engineering of EVs for targeted delivery [88]. (**b**) (**Left**) Post-isolation loading method. EVs are produced by unmodified parent cells. The components of the CRISPR-Cas9 system are loaded into EVs after their isolation and purification. (**Right**) Pre-isolation loading method. The components of the CRISPR-Cas9 system are transfected into parent cells. EVs are harvested and will contain the exogenous nucleic acid material by the natural EV production route [93].

To overcome targeting limitations, recent studies have explored modifying the EV surfaces to improve CRISPR-Cas9 delivery. Major approaches include the direct chemical modification of surface proteins and engineering of EV-secreting cells, as shown in Figure 4a [88]. In direct modification strategy, surface proteins on EVs can be modified using conjugation reactions, hydrophobic insertions, and receptor-ligand binding to enable targeted delivery. These methods are promising in cancer treatment, allowing precise cancer cell targeting by adding tumor-specific peptides to the EV surface. For example, Jia et al. successfully loaded superparamagnetic iron oxide nanoparticles (SPIONs) and curcumin into EVs and conjugated a neuropilin-1-targeting peptide (RGE) to the EV membrane, effectively targeting glioblastoma [89]. Alternatively, engineering EV-secreting cells involves modifying cells to produce EVs with specific surface molecules. For example, Bai et al. demonstrated that transfecting HEK293T cells with tLyp-1-lamp2b led to EVs with high specificity for tumor and lung cancer stem cells. These engineered EV, carrying siRNA, suppressed target genes and inhibited cancer growth effectively [90].

Another challenges, rapid clearance of EVs by immune cells, can be mitigated by strategies that enhance their stability and circulation time. For instance, integrin protein CD47, a “do not eat me” signal protein, onto the EV surface phagocytosis by macrophages, Similarly, PEGylation of EVs has been shown to enhance their molecular stability and prolong their circulation time, thereby increasing in vivo delivery efficiency [91].

EVs are ideal delivery vehicles for CRISPR-Cas9-based gene therapeutics because of their ability to carry various biomacromolecules, natural targeting capabilities, immune evasion, and the possibility of engineering modifications. These attributes are expected to play significant roles in clinical applications [92].

#### 3.2.2. Advantages of Extracellular Vesicles in CRISPR-Cas9 Delivery

EVs offers significant advantages as delivery vehicles for CRISPR-Cas9. They can stably encapsulate CRISPR-Cas9 components through two main approaches: pre-isolation and post-isolation loading methods, as shown in Figure 4b [93]. The pre-isolation loading method involves introducing genetic materials such as CRISPR-Cas9 into the parent cells, which naturally incorporate these molecules into their secreted EVs. This approach preserved the intrinsic structure and characteristics of EVs, ensuring stable CRISPR-Cas9 system [94]. On the other hand, the post-isolation loading method involves directly incorporating CRISPR-Cas9 into isolated EVs, offering greater flexibility and standardization across different cell types without requiring manipulation of the parent cells. These versatile loading methods, combined with EVs’ inherent targeting capability and biocompatibility, highlight their potential as effective tools for CRISPR-Cas9-based gene therapy [95].

One innovative example of EV-based delivery is the genome editing delivery system (GEDEX), a platform designed to enhance CRISPR-Cas9 delivery efficiency and precision. GEDEX leverages HEK293 cells engineered to overexpress Cas9 and sgRNA, producing EVs loaded with these components for targeted delivery. This system, which effectively induces DNA double-strand breaks in specific genes, such as MYD88, has been shown to successfully edit genes like Ptgs1 in various tissues—including the kidneys, brain, spleen, heart, lungs, and liver—in mouse models [96]. GEDEX represents a promising advancement, showcasing the potential of EVs to deliver CRISPR-Cas9 for precise and efficient genome editing with broad clinical applications.

Despite these promising developments, challenges remain in ensuring the safety, consistency, and efficiency of EV-based CRISPR-Cas9 delivery. For example, the effects of biomacromolecules encapsulated during EV biogenesis on cellular functions require further investigation. Additionally, tumor-derived EVs pose potential risks, such as promoting tumor growth and metastasis, underscoring the need for rigorous safety evaluations and careful sourcing [97]. Efforts to overcome these challenges include exploring hybrid systems to enhance the loading efficiency and capacity of CRISPR-Cas9 RNPs within EVs [98].

### 3.3. Polymer-Based Nanoparticles

#### 3.3.1. Structure and Characteristics of Polymer-Based Nanoparticles

Polymeric nanoparticles are structures composed of monomers that serve as versatile chemical platforms capable of achieving a wide range of physicochemical properties by adjusting their size, structure, and functionality. These nanoparticles self-assemble into different structures through covalent and non-covalent interactions [99] can incorporate auxiliary components like lipids or polymers to enhance stability, efficacy, and tissue-targeting properties [100].

Their customized design makes these polymeric nanoparticles highly effective for delivering the CRISPR-Cas9 system, particularly for cell- and organ-specific targeting [101]. They stabilize and deliver large negatively charged molecules, like nucleic acids, through electrostatic interactions. These polymeric nanoparticles promote cellular uptake, mainly via clathrin-mediated endocytosis, enabling efficient intracellular delivery and gene editing [102].

#### 3.3.2. Advantages of Polymer-Based Nanoparticles in CRISPR-Cas9 Delivery

Polymeric carriers for improving CRISPR-Cas9 delivery efficiency and stability commonly include polyethyleneimine (PEI), chitosan, and dendrimers.

**Figure 5:**
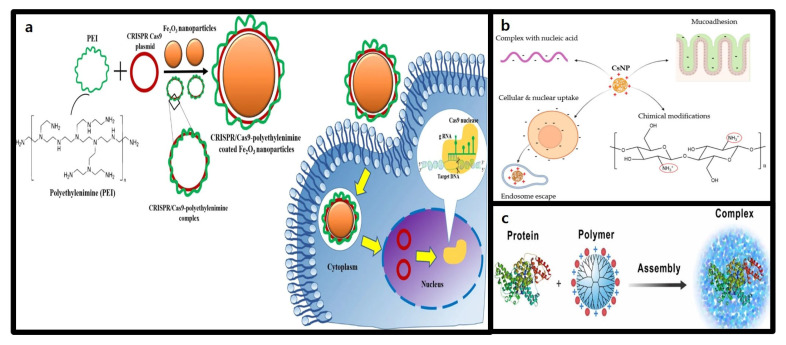
Representative polymer carriers for CRISPR-Cas9 system delivery. (**a**) Structure and intracellular delivery mechanism of nanocomplexes formed by the conjugation of PEI and CRISPR-Cas9 plasmid [113]. (**b**) Characteristics of chitosan nanoparticles (CsNP) and their advantages in CRISPR-Cas9 system delivery [106]. (**c**) Structure of dendrimers and their mechanism of interaction with proteins [109].

PEI, a cationic polymer with a high positive charge density, binds nucleic acids via electrostatic interactions, condensing them for efficient transfection (Figure 5a) [103]. Both linear and branched forms of PEI demonstrate excellent transfection efficiency in vitro and in vivo. For example, a report by Ryu et al. showed 25-kDa branched PEI (bPEI-25k) successfully delivered CRISPR-Cas9 plasmid DNA into Neuro2a cells, achieving over 70% transfection efficiency and more than 20% indel rates, comparable to Lipofectamine 2000 [104]. However, the high positive charge density of PEI can cause cytotoxicity, limiting clinical applications. To address this issue, modified PEIs with lower toxicity have been developed. O’Keeffe Ahern et al. demonstrated the use of highly branched poly(β-amino ester) polymers for delivering CRISPR-Cas9 RNPs, achieving indel rates of 15–20% in HEK293 cells and over 40% in recessive dystrophic epidermolysis bullosa (REDB) cells while maintaining over 72% cell viability [105].

Chitosan is a nontoxic, biodegradable natural polysaccharide obtained through the N-deacetylation of chitin that exhibits excellent biocompatibility [106]. It is composed of repeating units of D-glucosamine and N-acetyl-D-glucosamine, where the D-glucosamine units carry a positive charge at a specific pKa (6.5) or lower. This positive charge of D-glucosamine facilitates the formation of complexes between CsNPs and negatively charged nucleic acids, enhances mucoadhesion, promotes cellular and nuclear uptake, and enables endosomal escape. Moreover, D-glucosamine allows for various chemical modifications to improve the chemical properties of chitosan. As illustrated in Figure 5b, these advantages enable the creation of nanoparticles that form a highly efficient delivery system. However, low transfection efficiency is pointed out as the biggest drawback. Chemical modifications involving conjugation with 5β-cholic acid, deoxycholic acid, or stearic acid have been applied to enhance interactions with cell surfaces and improve the transfection efficiency, making chitosan a suitable polymer carrier for CRISPR-Cas9 delivery [107]. Liu et al. designed dual-targeting polymer-inorganic hybrid nanoparticles combining with protamine sulfate, calcium carbonate, calcium phosphate, and carboxymethyl chitosan. These nanoparticles successfully delivered CRISPR-Cas9 for CDK11 gene knockout in tumor cells, achieving effective nuclear delivery and gene editing [108].

Dendrimers, hyperbranched nanopolymeric structures, can extend radially from a central core and feature a three-part architecture: a central core, an interior structure of repetitive branches, and an outer shell with terminal functional groups [109]. mThis unique 3D geometry allows for precise and efficient drug delivery, combining characteristics that support diverse therapeutic strategies (Figure 5c). Dendrimers can interact with proteins through cationic (amine, imidazole, and guanidine) and anionic (carboxylate) surface groups, facilitating high-affinity binding to RNPs and enabling effective delivery of the Cas9 protein to various cell lines. Consequently, the use of dendrimers has been shown to significantly enhance the CRISPR-Cas9 editing efficiency [110].

Polyamidoamine (PAMAM) dendrimers are widely used as highly branched polymers for gene delivery vectors. Liu et al. demonstrated that modifying PAMAM with phenylboronic acid enhances its utility as a Cas9 protein delivery platform [109]. While unmodified PAMAM primarily interacts with negatively charged plasmid DNA through its amine groups, modification with phenylboronic acid enables residual amine groups to bind with anionic protein groups via electrostatic interactions. This modification allows boronic acid-rich dendrimers to form uniform nanoparticles with proteins of diverse sizes and ioelectric points (pIs), preserving the biological activity of delivered proteins after intracellular release. Notably, the modified dendrimer successfully complexed with both Cas9 protein and sgRNA, facilitating effective delivery of RNPs to various genomic loci in different cell lines. Compared to the cationic lipid-based Cas9 delivery vector CRISPRMAX (Invitrogen, Carlsbad, CA, USA), this platform showed superior editing efficiency across multiple target genes. However, further studies are needed to evaluate the long-term toxicity and metabolic byproducts of boronic acid derivates in vivo before clinical application. This study highlights the potential of boronic acid-rich dendrimers as efficient and versatile platforms for advancing CRISPR-Cas9-based gene therapeutics.

Challenges that CRISPR-Cas9-based gene therapeutics can face are immune responses and in vivo accumulation. Polymer-based nanoparticles, made from biocompatible materials, can address these issue. Coating the nanoparticle surface with polyethylene glycol (PEG) enhances immune evasion and prolongs circulation time, improving in vivo stability [111]. Additionally, biodegradable polymers, such as polylactic acid (PLA), polyglycolic acid (PGA), and their copolymer PLGA, naturally degrade in the body, reducing toxicity and preventing in vivo accumulation, ensuring both safety and efficiency [112].

### 3.4. Gold Nanoparticles

#### 3.4.1. Structure and Characteristics of Gold Nanoparticles (AuNPs)

AuNPs are notable for their unique optical properties, versatility in controlling size, shape, and charge, and advantages such as strong binding affinity, prolonged circulation half-life, and rapid uptake by tumor cells [114]. Surface functionalization of AuNPs with various ligands further enhances their stability, biocompatibility, and targeting capabilities [115]. Their ability to load a wide range of molecules, including small-molecule drugs, peptides, proteins, and DNA, is a critical factor enhancing their value as delivery vehicles for CRISPR-Cas9 systems [116].

Rotello et al. developed arginine-functionalized gold nanoparticles (ArgNPs) for the efficient delivery of negatively charged RNPs. A polyglutamate peptide tag to the N-terminus of Cas9 increased the charge density, while a nuclear localization signal (NLS) protein attached to the C-terminus facilitated nuclear entry. The length of the glutamate (E) tag significantly influenced nanocomplex size, cellular uptake and delivery efficiency. Notably, increasing the E-tag length from E0 to E20 improved the cytoplasmic delivery of Cas9En, with the nanocomplex releasing Cas9E20 efficiently into nucleus post-endocytosis. Gene-editing experiments using Cas9E15-RNP/ArgNPs achieved a knockout efficiency of 30% for PTEN and AAVS1 in HeLa cells [117]. This study highlighted the potential of AuNPs as robust platforms for gene therapeutics, particularly in enabling efficient and precise CRISPR-Cas9-based gene editing applications.

#### 3.4.2. Advantages of Gold Nanoparticles in CRISPR-Cas9 Delivery

Many gene therapies for genetic disorders rely on HDR-based approaches, but achieving efficient HDR in vivo remains a challenge [118]. Lee et al. developed AuNP-based CRISPR-Cas9 delivery system to address this need. In their study, 15 nm AuNPs were functionalized with single-stranded DNA modified with a 5′ thiol group to bind donor DNA templates via complementary base pairing. These AuNPs were conjugated with Cas9-RNP complexes, forming an AuNP-DNA-Cas9-RNP structure for efficient delivery to primary and stem cells. HDR experiments using HEK293 cells expressing blue fluorescent protein (BFP) showed that combining CRISPR-AuNPs with a donor oligonucleotide targeting the BFP gene induced GFP expression in 11.3% of cells. In primary myoblasts from Duchenne muscular dystrophy (DMD) model mice, HDR efficiency reached approximately 3%, outperforming Lipofectamine-based delivery (<1%) with reduced toxicity. In vivo studies demonstrated that intramuscularly CRISPR-AuNPs injection corrected the dystrophin gene and improved muscle function in mdx mice, underscoring the potential of AuNP-based CRISPR-Cas9 systems for treating genetic disorders [119].

**Figure 6:**
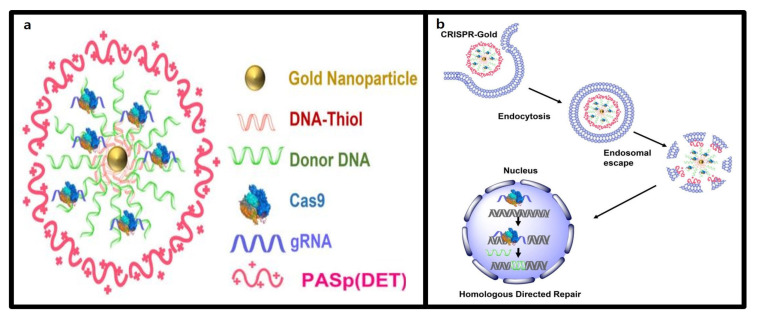
Efficient CRISPR-Cas9 delivery utilizing the structural advantages of AuNPs. (**a**) CRISPR-AuNPs are conjugated to thiol-modified oligonucleotides (DNA-Thiol), hybridized with single-stranded donor oligonucleotides, and subsequently complexed with Cas9 RNP and the endosomolytic polymer PAsp(DET), where “DET” denotes diethylenetriamine. (**b**) CRISPR-Gold is internalized into cells via endocytosis, induces endosomal disruption, and releases Cas9 RNP and donor DNA into the cytoplasm [119].

AuNPs also show promise for neurological applications, where targeted therapies are crucial due to the brain’s complex neural architecture. As shown in Figure 6, Lee et al. used DNA conjugated AuNPs and Cas9-RNP complexes coated with PAsp (DET) polymers to enhance endosomal escape and achieve precise brain-region delivery. This system effectively penetrated neurons, astrocytes, and microglia, enabling efficient gene editing [119]. The approach was applied to Fragile X syndrome (FXS), a genetic disorder caused by mutations in the FMR1 gene. Suppression of Grm5, which regulates excessive mGluR5 signaling linked to FXS pathology, reduced neural overactivation by 40–50% at the mRNA and protein levels. This localized suppression in the striatum improved behaviors such as repetitive actions in FXS mouse models, demonstrating that selective neural pathway targeting may suffice for autism treatment [120].

Despite these promising findings, concerns remain about the long-term safety of Au NPs. Following intravenous (IV) injection, AuNPs primarily accumulate in clearance organs such as the liver and spleen and are sequestered by macrophages within lysosomes [121]. This bioaccumulation raises potential toxicity concerns, particularly for therapies requiring repeated administrations. Addressing these challenges is critical for the clinical translation of AuNPs as effective gene therapy delivery platforms.

### 3.5. Mesoporous Silica Nanoparticles (MSNs)

#### 3.5.1. Structure and Characteristics of Mesoporous Silica Nanoparticles (MSNs)

**Figure 7:**
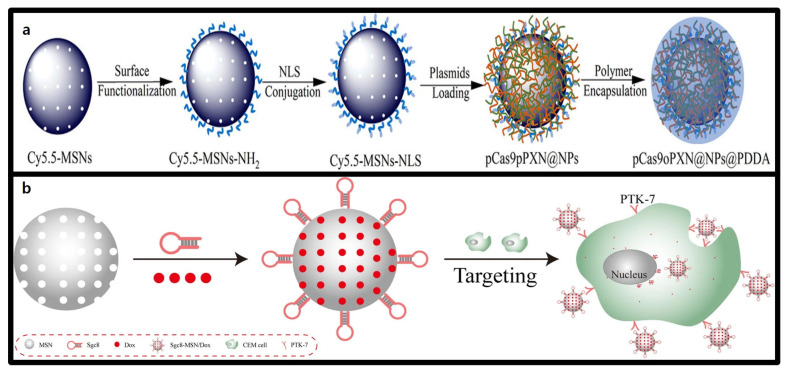
Schematic of functionalized MSN-based nanocarriers for CRISPR-Cas9 delivery. (**a**) The surface functionalization of MSNs with amino groups (-NH_2_) and nucleus-targeting sequences (NLS) facilitates the transport of CRISPR-Cas9 plasmids to the nucleus, providing effective protection and defense against degradation [123]. (**b**) The surface of the MSNs is functionalized with Sgc8 aptamers and loaded with the anticancer drug doxorubicin (Dox). This configuration allows for selective targeting of the CEM cells, which overexpress the PTK-7 receptor. Upon binding to the target cells, the system is designed to deliver the drug to the nucleus, thereby exerting therapeutic effects [131].

MSNs are promising candidates for delivering therapeutic genetic materials, particularly CRISPR-Cas9 systems. Their unique properties—such as high surface area, tunable pore size, customizable particle size, and surface functionalization—enable the stable loading and delivery of large genetic material, making them highly applicable for genetic therapeutics [122]. Functionalizion with ligands, cationic polymers, or peptides, as shown in Figure 7a, further enhances their cellular uptake and transfection efficiency, expanding their potential application [123].

The surface functionalization of MSNs is crucial in determining their gene delivery efficiency. Unmodified MSNs possess negatively charged silanol groups, which hinder interactions with the negatively charged nucleic acids [124]. To address this issue, introducing positive charges via amination or coating with cationic polymers is a common strategy. For instance, MSNs functionalized with polyethylenimine (PEI) provide positive charges that enhance electrostatic interactions with nucleic acids, effectively condensing and protecting DNA. Additionally, PEI promotes endosomal escape, leading to enhanced gene expression and low cytotoxicity, establishing these MSNs as effective CRISPR-Cas9 delivery vectors [125].

Another significant feature of MSNs is the ability to adjust pore structures, which is critical for controlling the loading and release of genetic therapeutics. Enlarged pore sizes accommodate large molecules like CRISPR-Cas9 but may cause burst release [126]. To address this issue, non-covalent binding methods have been explored. For example, nickel-functionalized MSNs loaded with His-tagged β4 proteasome subunits (29 kDa) within 25–30 nm pores showed effective delivery and degradation of overexpressed tau protein in HEK293 cells. Tau protein aggregation disrupts intercellular signaling a primary pathological feature of Alzheimer’s disease. This system outperformed conventional proteasomes in reducing tau levels, showcasing the potential of MSN-based therapeutics for protein misfolding disorders [127].

MSNs also possess structural stability and biodegradability in vivo. Their degradation products, such as silicic acid, are biocompatible, reducing the risk of in vivo accumulation. However, surface damage in aqueous environments, especially in the presence of amines, poses a challenge. Functionalization methods such as 3-aminopropyl(triethoxy)silane (APTES) [128], and zwitterionic modifications [129] prevent degradation, particle contaminations and aggregation in biological systems. These attributes make MSNs ideal carriers for nanomedicine, capable of minimizing toxicity and immune responses while offering robust structural integrity for delivering therapeutics.

#### 3.5.2. Advantages of Mesoporous Silica Nanoparticles (MSNs) in CRISPR-Cas9 Delivery

The surface functionalization of MSNs significantly improves their targeting capabilities, thereby addressing one of the primary challenges associated with CRISPR-Cas9 systems—off-target effects [130]. A notable strategy for this enhancement is aptamer-based surface functionalization. Aptamers are short, single-stranded oligonucleotides (2–80 nucleotides) that bind to specific target molecules with high specificity and affinity. When conjugated with MSNs, aptamers facilitate the internalization of therapeutic agent into target cells, enabling tissue-specific delivery. This approach not only reduces off-target effects but also addresses the low efficiency often observed with non-viral delivery systems. For example, Yang et al. developed an MSN-PEM-based aptamer nanocarrier using the DNA aptamer sgc8, as shown in Figure 7b, which demonstrated high cell recognition and drug delivery efficiency in cancer cells [131]. This highlights the dual advantages of MSN surface modification: improved gene delivery efficiency and increased applicability for CRISPR-Cas9-based therapeutics through targeted delivery and controlled release.

MSNs are also gaining attention as multifunctional platforms for the simultaneous delivery of CRISPR-Cas9 systems and small-molecule drugs, a strategy that holds promise for addressing complex diseases. Fernández et al. developed a multifunctional MSN platform capable of co-delivering anti-inflammatory drugs VX-765 and CRISPR-Cas9. This system successfully edited the Gasdermin D gene, reducing inflammation-induced cell death and amplifying anti-inflammatory effects [132]. These results highlight the potential of MSNs for co-delivery applications, maximizing therapeutic efficacy by integrating gene editing and pharmacological interventions.

The porous structure of MSNs facilitates the development of theranostic systems that combine therapy and diagnostics. This “theranostics” approach integrates treatment with various imaging modalities, including MRI, photoacoustic (PT) imaging, near-infrared fluorescence (NIRF), PET, SPECT, and CT (X-ray), enabling precise lesion identification while delivering therapeutics [133]. MSN-based theranostic platforms can be customized to optimize the advantages of each therapeutic and imaging agent, offering a tailored approach to personalized medicine. These MSN-based gene therapeutics are particularly promising for image-guided cancer diagnosis and treatment, underscoring their potential in precision medicine [134].

Despite these promising advancements, several challenges must be addressed before MSN-based CRISPR-Cas9 delivery systems can be clinically applied. Ensuring long-term stability, in vivo distribution, and biodegradability of MSN is crucial. Moreover, comprehensive validation using appropriate preclinical models and the establishment of regulatory guidelines are essential for transitioning these platforms from research to clinical settings [135].

## 4. Stimuli-Responsive Nanoformulations

Despite research advancements and achievements in non-viral CRISPR-Cas9 delivery systems, critical challenges such as low efficiency and off-target effects remain unresolved. Nonviral delivery systems face efficiency limitations owing to multiple physiological barriers [136]. The plasmid DNA, mRNA, and RNP forms of CRISPR-Cas9 are negatively charged, resulting in electrostatic repulsion with the negatively charged cell membrane, thereby reducing the cellular uptake efficiency. If these barriers are not overcome, even if the nanoparticles successfully enter the cell, they may be degraded within endosomes or exhibit limited diffusion, ultimately lowering the success rate of gene editing [137]. Regarding off-target effects, although CRISPR-Cas9 is designed for the precise editing of specific gene sequences, its targeting may not be adequately controlled in the complex biological environment. Moreover, the current nanoparticle-based delivery systems may lead to nonspecific distribution in normal tissues, which could increase the risk of unintended genetic mutations or toxic reactions [138].

Stimuli-responsive nanoformulations have gained attention because of their ability to address these challenges [139]. As shown in Figure 1b, stimuli-responsive nanoformulations are drug delivery systems that selectively activate and release the CRISPR-Cas9 system in response to internal or external stimuli. These systems are designed to activate the CRISPR-Cas9 system only under specific physiological conditions or stimuli, enabling the precise spatiotemporal control of gene editing [140]. Stimuli-responsive nanoformulations can finely regulate the release of CRISPR-Cas9 in response to internal triggers, such as pH, enzymes, ATP, or redox states, as well as external stimuli, such as light, ultrasound, or magnetic fields [141]. Despite being non-viral vectors, to emphasize this content, it was decided to separate it into its own chapter for detailed explanation. Therefore, they play a crucial role in minimizing side effects and enhancing in vivo safety for gene therapy [142].

### 4.1. Internal Stimuli-Responsive Nanoformulations

Internal stimuli-responsive nanoformulations are innovative delivery systems that selectively activate CRISPR-Cas9-based gene therapeutics in response to pathological changes or internal signals within the body. This technology was designed to detect microenvironmental differences between normal and pathological tissues, ensuring that gene editing operates only at specific sites, thereby improving accuracy, efficiency, and safety [143].

**Figure 8:**
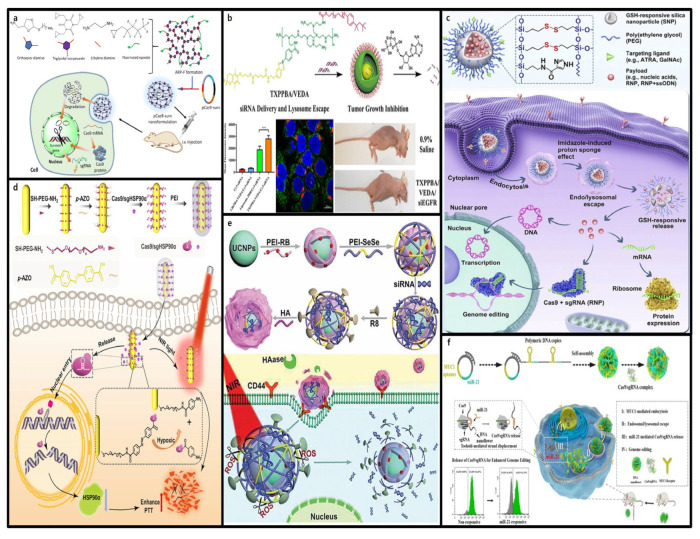
Overview of internal stimuli-responsive nanoformulations. (**a**) pH-responsive intracellular CRISPR-Cas9 delivery strategy [150]. (**b**) ATP-triggered boronate bond release of siRNA in cancer cells [154]. (**c**) CRISPR-Cas9 delivery system and activation mechanism based on GSH-responsive silica nanoparticles (SNPs) [157]. (**d**) The hypoxia-responsive CRISPR-Cas9 system is designed to reduce tumor thermal tolerance and facilitate the delivery of Cas9-sgHSP90α to the nucleus for gene editing [162]. (**e**) An enzyme-responsive carrier that releases siRNA by degrading nanoparticles using hyaluronic acid (enzyme) [169]. (**f**) Schematic diagram of microRNA-responsive DNA nanoflower for release of Cas9/sgRNA and enhanced genome editing [171].

Internal stimuli-responsive nanoformulations function under various physiological conditions such as pH, ATP levels, redox states, hypoxic environments, enzymes, and RNAs (Figure 8). Notably, these systems can operate effectively under pathological conditions such as cancer and can be functionalized with bioresponsive moieties on the surface of the delivery vehicle to enhance the multifunctionality of the CRISPR-Cas9 system [144].

#### 4.1.1. pH-Responsive CRISPR-Cas9 Delivery

Various environments within the body exhibit unique pH levels, which play a crucial role in the design of bioresponsive drug delivery systems [145]. pH-responsive nanodelivery systems leverage these pH variations and are used as an effective strategy for diagnosing and treating infections and malignant tumors, as well as the delivery of CRISPR-Cas9 systems. During the delivery process, endosomes and lysosomes act as major intracellular barriers, and their acidic environments pose the risk of rapid degradation of CRISPR-Cas9 components. Therefore, pH-responsive nanoformulations have been developed to prevent this phenomenon and promote efficient endosomal escape [146].

Imidazole is one of the most widely used pH-responsive agents owing to its alkaline nitrogen atoms, which induce a proton sponge effect that facilitates endosomal escape. In the acidic environment of endosomes (pH < 6.5), protons are absorbed by the alkaline nitrogen atoms (pH 6.5–6.9), leading to the influx of chloride ions and water to balance the endosomal membrane’s charge. This osmotic pressure results in endosomal rupture, allowing the release of cargo such as the CRISPR-Cas9 system into the cytoplasm [147]. Wang et al. designed solid lipid nanoparticles (SLNs) decorated with cell-penetrating peptides for the delivery of CRISPR-Cas9 plasmids and sgRNA. This system (HuR/CRISPR SLN-HPR) included pH-sensitive H-peptides that transition to hydrophilic states in the tumor microenvironment (pH 6.0–6.5). This transition is induced by the protonation of imidazole groups, which exposes hidden P-peptides, enabling the targeting of the epidermal growth factor receptor (EGFR) in SAS cells. Additionally, R-peptides facilitate nuclear targeting for the delivery of the CRISPR-Cas9 system within cells. This technology promotes payload release in the tumor microenvironment, enhancing the efficacy of epirubicin chemotherapy by eliminating HuR, which contributes to cancer cell survival and resistance [148].

Other pH-responsive cationic polymers have been used to deliver CRISPR-Cas9. For example, pH-sensitive amphiphilic polymer micelles such as poly{(1,4-butanediol) diacrylate-β-N,N-diisopropylethylenediamine}-PE remain stable at neutral pH and in the extracellular environment of tumor cells but release paclitaxel and Akt siRNA in the acidic lysosomal environment. This leads to the suppression of Akt and P-glycoprotein 1 expression, thereby promoting apoptosis in breast cancer cells [149].

Moreover, the protonation of specific chemical groups in an acidic environment can induce hydrophobic-to-hydrophilic transitions or charge reversal of nanoparticles, facilitating their degradation and payload release. Qi et al. utilized this one-pot ring-opening polymerization method to design fluorinated acid-responsive polycations (ARP-Fs) [150]. (Figure 8a) These polycations form stable ARP-F/pCas9-surv nanoparticles through electrostatic interactions with negatively charged plasmids and remain relatively stable under neutral pH owing to the orthoester linkages. However, in acidic environments, these linkages are cleaved, releasing the pCas9-surv plasmid. Notably, when accumulated in the mildly acidic environment of A549 lung cancer cells, orthoester bond cleavage released the pCas9-surv plasmid encoding the Cas9 protein and sgRNA, efficiently targeting and disrupting the intended gene. Furthermore, the elimination of the survivin gene enhanced the sensitivity of cancer cells to antitumor drugs such as temozolomide, offering an effective combination therapy for cancer treatment.

#### 4.1.2. ATP-Responsive CRISPR-Cas9 Delivery

ATP is the primary energy source for living cells, and its concentration in tumor tissues is approximately 1000 times higher than that in normal tissues [151]. This significant difference presents great potential for the design of ATP-responsive nanoformulations for CRISPR-Cas9 delivery and activation.

Yang et al. proposed ATP-responsive Zeolitic Imidazolate Framework-90 (ZIF-90) as a platform for cytosolic protein delivery and CRISPR-Cas9 genome editing. Imidazole-2-carboxaldehyde and Zn^2+^ self-assemble with proteins to form ZIF-90-protein nanoparticles, effectively encapsulating proteins during the process. Due to these structural characteristics, in the presence of ATP, competitive coordination between ATP and Zn^2+^ in ZIF-90 leads to the degradation of ZIF-90-protein nanoparticles. Through this process, the CRISPR-Cas9 system is released into target cells, and it was demonstrated that ZIF-90-protein nanoparticles can deliver a variety of proteins into the cytosol, regardless of protein size or molecular weight [152]. This study highlights that intracellular ATP levels can determine the delivery efficiency of the CRISPR-Cas9 system, thereby offering new gene therapy opportunities for neurogenetic disorders, such as Alzheimer’s disease or schizophrenia, which are caused by abnormal ATP production. Moreover, this ATP-responsive may also be used for the coordinated delivery of chemotherapeutic drugs and siRNA.

For example, cationic polyplex micelles derived from 4-carboxy-3-fluorophenylboronic acid, whose surfaces are protected by PEG to enhance their in vivo stability, have been developed. d-Gluconamide within the micelles responds to increased intracellular ATP concentrations, promoting the de-condensation of pDNA, thereby enhancing gene transfection efficiency [153]. Another example is the use of Triton X-100, a nonionic surfactant known for its high membrane permeability. As shown in Figure 8b, Triton X-100 significantly improves cellular uptake and the endosomal/lysosomal escape of siRNA. ATP-responsive nanocarriers functionalized with Triton X-100 have been shown to effectively deliver siRNA to cancer cells and successfully suppress tumor growth in mouse models [154].

#### 4.1.3. Redox-Responsive CRISPR-Cas9 Delivery

The redox potential difference between intracellular and extracellular environments is considered an ideal factor for designing CRISPR-Cas9-based gene therapeutic delivery systems. Specifically, within cells, the reducing agent glutathione (GSH) is present at concentrations two to three times higher than those in the extracellular fluid, creating a favorable environment for the design of intracellular delivery systems [155]. The most critical component of redox-responsive delivery systems is the disulfide bond, which remains stable under low reductive conditions, such as in blood circulation or the extracellular environment, but is reduced to sulfhydryl groups in a highly reductive intracellular environment, leading to decomposition [156].

Using this principle, Wang et al. synthesized GSH-responsive silica nanoparticles (SNPs). As shown in Figure 8c, disulfide crosslinkers were incorporated into the silica structure, allowing GSH-responsive cargo release when internalized by the target cells. Imidazole-containing components have been introduced to enhance the endosomal escape capabilities. SNPs demonstrate high efficiency and biocompatibility in delivering various cargo, including the CRISPR-Cas9 system, and the surfaces of SNPs can be functionalized with different targeting ligands. In vivo studies have shown that SNPs conjugated with all-trans-retinoic acid (ATRA) and injected into the subretinal space successfully delivered mRNA and RNP to retinal pigment epithelial (RPE) cells in mice. Similarly, SNPs conjugated with GalNAc and administered intravenously effectively delivered mRNA and RNP to liver cells, achieving efficient genome editing [157].

Chen et al. developed a GSH-degradable polymeric nanocapsule system to deliver CRISPR-Cas9 RNPs. This RNP nanocapsule was formed by crosslinking a mixture of cationic and anionic monomers in situ using N,N′-bis(acryloyl)cystamine, a GSH-degradable crosslinker, to encase the Cas9 RNP through disulfide bonds. These bonds were readily cleaved at high GSH concentrations in the cytoplasm, leading to the decomposition of the nanocapsule shell and the release of RNPs, enabling gene editing. This RNP nanocapsule system demonstrated excellent biocompatibility and maintained its stability even after lyophilization. Strong gene-editing capabilities were demonstrated through in vitro experiments in HEK293 cells, T cells, and stem cells, as well as in vivo experiments targeting retinal pigment epithelial tissue and skeletal muscle in Ai14 mice [158].

Beyond the intracellular reducing conditions, the concentration of reactive oxygen species (ROS) increases in various pathological conditions such as cancer, stroke, atherosclerosis, and tissue damage. Notably, tumor cells have significantly higher ROS levels than normal cells, making ROS a viable factor for CRISPR-Cas9-based gene therapy delivery strategies [159]. ROS are highly reactive molecules, or free radicals, and these properties form the basis for the more efficient activation and release of the CRISPR-Cas9 system in ROS-rich environments.

For example, Yan et al. developed a CRISPR-Cas9 prodrug nanosystem (NanoProCas9) capable of specifically activating gene editing in inflammatory environments. First, cationic poly(β-amino ester) (PBAE) was used to form a complex with plasmid DNA encoding destabilized Cas9 (dsCas9) containing dihydrofolate reductase (DHFR) domains. Then, the PBAE/plasmid complex was coated with a macrophage membrane, enabling the CRISPR-Cas9 system to be targeted and delivered to inflammatory tissues. Finally, the ROS-responsive precursor molecule, trimethoprim (TMP), was introduced into the macrophage membrane via lipid fusion. In normal environments, structurally unstable dsCas9 induced by NanoProCas9 is easily degraded by ubiquitin-dependent proteasomes. However, in inflammatory environments with high ROS levels, TMP is released, stabilizing dsCas9 and enabling effective gene editing. This ROS-dependent NanoProCas9 system responded to elevated ROS levels in a dextran sulfate sodium (DSS)-induced colitis model in BALB/c mice, achieving efficient CRISPR-Cas9 delivery and targeted activation for genome editing. Furthermore, when ROS levels return to normal after treatment, NanoProCas9 loses its gene-editing capability, offering a safer strategy for the in vivo application of CRISPR-Cas9 systems [160]. These findings suggest that ROS-responsive CRISPR-Cas9 delivery systems could also be actively utilized in the treatment of other inflammatory diseases, such as liver and lung injuries, atherosclerosis, and stroke.

#### 4.1.4. Hypoxia-Responsive CRISPR-Cas9 Delivery

Hypoxia is a pathological feature associated with numerous diseases, particularly solid tumors, in which rapid cell proliferation and vascular dysfunction lead to reduced oxygen supply, creating a hypoxic tumor microenvironment [161]. This condition has significant potential for designing hypoxia-responsive nanoformulations for CRISPR-Cas9 delivery and activation. Notable hypoxia-responsive materials include the azobenzene complexes and 2-nitroimidazole.

Recently, utilizing azobenzene complexes, Li et al. designed a hypoxia-responsive gold nanorod (AuNR)-based CRISPR-Cas9 nanocarrier for the knockout of the heat shock protein 90α (Hsp90α) gene. This carrier was constructed using Cas9-sgRNA conjugated to AuNRs via the hypoxia-sensitive linker azobenzene-4,4′-dicarboxylic acid. In the hypoxic microenvironment of tumor cells, an imbalance in the redox state increases the reductive stress, reducing the azobenzene-4,4′-dicarboxylic acid linker and releasing Cas9-sgRNA from the AuNRs. Consequently, the knockout of the Hsp90α gene enhanced the sensitivity of tumor cells to photothermal therapy mediated by AuNRs [162] (Figure 8d). This research highlights the potential of hypoxia-responsive nanocarriers to enable effective drug delivery and gene editing within hypoxic tumor cells, thereby maximizing therapeutic efficacy. And these hypoxia-responsive nanofomulations can be utilized as anticancer agents through the delivery of siRNA.

As a related example, a hypoxia-responsive nanoformulation modified with the hypoxia-sensitive moiety 2-nitroimidazole was developed. Kang et al. synthesized an amphiphilic bPEI1.8k-C6-NI polycation by conjugating the hydrophilic 2-nitroimidazole with alkylated polyethyleneimine (bPEI1.8k-C6). bPEI1.8k-C6-NI can self-assemble into micelle-like aggregates in aqueous solutions, enhancing the structural stability of the bPEI1.8k-C6-NI/siRNA polyplex and increasing its cellular uptake. Upon delivery to hypoxic tumor cells, this system undergoes selective nitro-to-amino reduction, resulting in structural changes. These reduction-induced changes loosen the polyplex structure, facilitating siRNA release into the cytoplasm, thereby improving the gene silencing efficiency. Consequently, survivin-targeted siRNA-loaded polyplexes demonstrated remarkable antitumor effects not only in hypoxic cells, but also in tumor spheroids and tumor-bearing mice. These studies suggest that hypoxia-responsive nanofomulations hold great potential for tumor-targeted therapy and can also be effectively utilized as CRISPR-Cas9-based gene therapeutics [163].

#### 4.1.5. Enzyme-Responsive CRISPR-Cas9 Delivery

Enzymes are essential components of biological metabolic processes and are known for their high selectivity and specificity. Enzyme-responsive CRISPR-Cas9 delivery systems have been developed owing to their specificity for particular substrates. Most enzyme-responsive nanodelivery systems use extracellular enzymes, and the mechanisms of drug release can be categorized into physical or chemical pathways [164]. Enzyme-responsive delivery systems are particularly useful in the tumor microenvironment, leveraging enzymes such as matrix metalloproteinases (MMPs) and hyaluronidase (HAase), which are overexpressed in tumor tissues. MMPs play crucial roles in regulating the microenvironment and signaling pathways during tumor progression and metastasis [165], whereas HAase degrades hyaluronic acid, contributing to tumor development [166].

Yin et al. developed an overcharged polypeptide (SCP) system coupled with Cas9-sgRNA, using a dithiocyclopeptide linker containing an MMP-2-sensitive sequence and disulfide bonds cleaved by GSH. This system is specifically degraded in the tumor microenvironment by the overexpression of MMP-2, releasing CRISPR-Cas9 RNP. Subsequently, the disulfide bonds are cleaved in the GSH-rich cytoplasm, activating the RNP and enabling gene editing. In HeLa cells, this system demonstrated a gene editing efficiency of approximately 31.9% [167].

Li et al. developed an HA-coated core-shell nano delivery system for targeted and efficient genome editing using CRISPR-Cas9. In this system, CRISPR-Cas9 plasmid was condensed by fluorinated polymer (PF33) to form a nanoscale core, and PF33 provided endosomal escape ability and high transfection efficiency. When delivered into tumor tissues, the HA coating was degraded by HAase, exposing the positively charged PF33-Cas9 core, which enhanced both the cellular internalization and nuclear delivery efficiency of CRISPR-Cas9. Notably, the MutT Homolog1 (MTH1) gene, which is overexpressed in tumor cells to prevent DNA damage and apoptosis, was effectively disrupted when delivered with Cas9-MTH1 plasmid in SKOV3 cells. Furthermore, in vivo experiments demonstrated that MTH1 was successfully targeted and disrupted, significantly inhibiting tumor growth [168]. This enzyme-responsive nanoformulation can also be utilized as an anti-cancer agent through the delivery of pDNA or siRNA.

For example, hyaluronic acid-based carriers targeting CD44 receptors have also been developed. CD44, the most common HA receptor for hyaluronic acid, is abundantly present on the cell membranes of inflammatory and cancer cells and plays an essential role in active targeting. Because of these characteristics, hyaluronic acid prevents the leakage of siRNA or pDNA during delivery and targets tumor cells overexpressing CD44 receptors. When multilayered upconversion nanoparticles (nano-onions) are coated with hyaluronic acid, the excessive HAase secreted by the tumors degrades the hyaluronic acid coating, allowing the nanocarrier to bind to CD44 receptors. This leads to cellular uptake by tumor cells, followed by the activation of a multi-stimuli-responsive system that fully degrades the nanocarrier and triggers the release of the drug payload [169] (Figure 8e). These studies indicate that enzyme-responsive nanoformulations can be effectively utilized as CRISPR-Cas9-based gene therapeutics and suggest their great potential for tumor-targeted therapy.

#### 4.1.6. RNA-Responsive CRISPR-Cas9 Delivery

RNA is abnormally expressed in pathological conditions, such as tumors, and contributes to tumor growth and metastasis [170]. RNA-responsive systems leverage this phenomenon to control the activation of Cas9 based on the presence of specific RNAs in target cells and tissues, primarily by utilizing microRNAs (miRNAs) or messenger RNAs (mRNAs). This approach enables highly precise gene editing within specific cells or tissues and is particularly effective in pathological environments such as cancer cells.

The miR-21-responsive Cas9/sgRNA RNP delivery system utilizing DNA nanoflowers (DNFs) plays a critical role in the efficient delivery of Cas9/sgRNA complexes. The DNFs were prepared by rolling circle amplification (RCA) using cyclized single-stranded DNA (ssDNA) templates and contained multiple copies of MUC1 aptamers and miR-21 binding sequences. The Cas9/sgRNA loaded onto the DNFs included an extended sequence 7 nt shorter than miR-21 to enable sequence hybridization, which incorporated the miR-21 binding sequence. Additionally, this system was functionalized with MUC1 aptamers to effectively promote lysosomal escape. The DNF/Cas9/sgRNA nanoformulation is internalized into cells through MUC1-mediated endocytosis, undergoes Mg^2+^-induced endosomal/lysosomal escape, and is subsequently delivered to the cytoplasm. In the cytoplasm, miR-21 triggers the release of the Cas9/sgRNA complex from the DNFs through a toehold-mediated strand-displacement mechanism. The released Cas9/sgRNA complex can bypass the karyotheca and be transported into the cell nucleus via nuclear localization signal (NLS) peptides fused to Cas9. This approach demonstrated effective gene editing both in vitro and in vivo in HeLa cells [171] (Figure 8f).

Li et al. designed a nanocarrier comprising DNA and multilocked DNA valves for mRNA-responsive drug delivery. Doxorubicin (Dox) was encapsulated within mesoporous nanoparticles and capped with two-gate DNAs via electrostatic interactions. These gate DNAs were complementary to the tumor-associated GT mRNA or Tk1 [172]. This study provides a promising platform for CRISPR-Cas9 systems and anti-cancer drug delivery through RNA-responsive delivery strategies in cells where miRNA or mRNA is overexpressed. Furthermore, by utilizing specific biomarkers, it enables tumor cell-specific gene editing, offering the potential to address off-target effects and safety issues associated with conventional CRISPR-Cas9-based gene therapies.

### 4.2. External Stimuli-Responsive Nanoformulations

**Figure 9:**
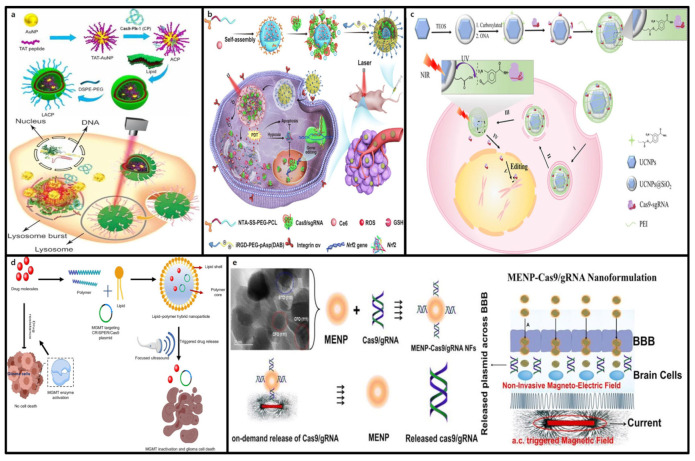
Overview of external stimuli-responsive nanoformulations. (**a**–**c**) are based on light-responsive CRISPR-Cas9 delivery. (**a**) A thermosensitive CRISPR-Cas9 release system was designed using AuNPs, leveraging the photothermal effect [178]. (**b**) A schematic of NIR-sensitive nanoparticles generating ROS through NIR irradiation and releasing Cas9/sgRNA via disulfide bond reduction [179]. (**c**) Design of the UCNP (upconversion nanoparticle)-based CRISPR-Cas9 delivery system for NIR light-controlled gene editing [180]. (**d**) A schematic of an ultrasound-responsive CRISPR-Cas9 delivery system loaded with temozolomide (TMZ) and CRISPR-Cas9 plasmid DNA targeting O6-methylguanine-DNA methyltransferase (MGMT) [183]. (**e**) A schematic of magnetic-responsive CRISPR-Cas9 delivery systems using MENPs [189].

External stimuli-responsive nanoformulations leverage external physical triggers to enable the precise control of the CRISPR-Cas9 gene-editing system. These systems use physical stimuli, such as light, ultrasound, and magnetic fields, to regulate the structure, activity, delivery, and release of CRISPR-Cas9, thereby maximizing the safety and efficacy of gene editing [173]. (Figure 9) Notably, these systems offer the advantage of temporal and spatial precision in gene editing at target tissues, playing a critical role in minimizing off-target effects and reducing side effects [174].

#### 4.2.1. Light-Responsive CRISPR-Cas9 Delivery

Light-responsive CRISPR-Cas9 delivery systems utilize light of specific wavelengths to release CRISPR-Cas9 from polymers, cationic liposomes, and AuNPs, enabling the precise spatiotemporal control of gene editing [175]. These technologies leverage ultraviolet (UV), visible, and near-infrared (NIR) light to meticulously regulate the release of drugs or nucleic acids, thus maximizing gene editing efficiency and accuracy [176]. Specifically, NIR-based systems can penetrate deeper into tissues, making them more effective for targeted applications, and ensuring greater biocompatibility and safety. Controlled CRISPR-Cas9 delivery and activation through light-responsive systems offers a noninvasive solution with high spatial and temporal precision, minimizing off-target effects and side effects [177]. This approach enhances the capabilities of traditional non-viral delivery systems and is recognized as a significant advancement in gene-editing technologies with increased accuracy and control.

The photothermal effect is one of the most widely employed mechanisms of light-mediated gene delivery. This process typically utilizes photothermal materials, such as AuNPs, which absorb specific light wavelengths to generate localized heat. This heat induces structural changes in nanoparticles disrupts chemical bonds, facilitating the release of the CRISPR-Cas9 system. Wang et al. designed a heat-sensitive CRISPR-Cas9 release system using lipid-encapsulating AuNPs. As shown in Figure 9a, cationic AuNPs were conjugated with a nuclear-targeting TAT peptide, and the negatively charged Cas9-sgPlk-1 plasmid (CP) was condensed onto the cationic AuNPs through electrostatic interactions, forming AuNP/CP complexes (ACP). They were then coated with lipid and lipid-PEG layers to form lipid-encapsulated LACP. Under 514 nm laser irradiation, the plasmon resonance of AuNPs within the LACP generated heat, triggering the photothermal effect and releasing the TAT/CP complex. The TAT peptide facilitated nuclear targeting, leading to the knockout of Plk-1 and induction of apoptosis in tumor cells [178]. This study demonstrated the efficacy of light-responsive systems in CRISPR-Cas9-based gene editing.

Photodynamic reactions activate photosensitizers using light to generate ROS, which can disrupt endosomal/lysosomal membranes or cleave ROS-sensitive chemical bonds to modulate payload release. This approach allows for the controlled release of nanoparticles containing CRISPR-Cas9, with tumor cells showing high sensitivity to ROS, enabling precise payload release in target cells. Deng et al. co-delivered CRISPR-Cas9 ribonucleoprotein (RNP) and the photosensitizer chlorin e6 (Ce6) to a nasopharyngeal carcinoma cell-implanted mouse model using near-infrared (NIR) light- and reduction-responsive polymeric nanoparticles. This delivery system encapsulated SpCas9/sgRNA RNP through repeated intravenous injections, inducing gene disruption targeting the key antioxidant regulator Nrf2 (nuclear factor erythroid 2-related factor 2) via the NHEJ mechanism. As shown in Figure 9a, after administration, NIR light irradiation at the tumor site generated ROS through Ce6, which induced cell apoptosis and disrupted endosomes/lysosomes, enabling the release of Cas9/sgRNA into the cytoplasm. In the cytoplasm, high levels of glutathione (GSH) cleaved the disulfide bond between nitrilotriacetic acid (NTA) and PEG, resulting in the separation of Cas9/sgRNA from the nanoparticles and the subsequent disruption of Nrf2 [179]. This study demonstrated that spatially controlled ROS generation through photodynamic reaction activates gene editing only in tumor tissues, effectively suppressing tumor cell metastasis and growth progression. Furthermore, the confirmation of the synergistic effect of photodynamic therapy and gene editing highlighted its potential as a promising approach for localized antitumor therapy.

Photon upconversion mechanisms absorb NIR light and convert it into visible light, thereby activating the CRISPR-Cas9 delivery systems. Upconversion nanoparticles absorb NIR light and emit visible light, which triggers the CRISPR-Cas9 system. This principle is based on the high tissue penetration of NIR light and the ability of visible light to effectively control the ion channels within the body [180]. As shown in Figure 9c, this system allows for the non-invasive control of CRISPR-Cas9 delivery and gene editing even in deep tissues, making it an attractive approach for selective gene editing. The upconversion mechanism addresses the challenge of low-energy NIR-induced photochemical reactions and utilizes visible light for surface-level applications on the skin and mucous membranes. Although UV, visible, and high-power NIR light carry the risk of thermal damage, co-delivery systems using ROS-degradable polycations and photosensitizers enable gene delivery at low light power densities, thereby enhancing safety and efficacy.

#### 4.2.2. Ultrasound-Responsive CRISPR-Cas9 Delivery

Ultrasound-responsive systems have emerged as pivotal tools in gene therapy for central nervous system (CNS) disorders. One of the most significant challenges in effective gene therapy for CNS diseases is crossing the blood-brain barrier (BBB) [181]. Conventional intracranial injections have limitations such as restricted gene expression and potential neural damage, whereas viral vectors often fail to cross the BBB effectively. In contrast, focused ultrasound (FUS)-mediated gene therapy has shown great potential for enabling the localized opening of the BBB for gene drug delivery, facilitating treatment strategies for CNS disorders [182].

Yang et al. used this approach to develop a PLGA core/cholesterol shell hybrid nanoparticle (LPHN) system capable of delivering a pCas9/O6-methylguanine-DNA methyltransferase (MGMT) plasmid. The system incorporated perfluoropropane (C3F8) to generate microbubbles, which were linked to the LPHNs via biotin–avidin binding. As shown in Figure 9d, FUS was employed to induce the vibration of the microbubble–LPHN complex, resulting in the opening of the BBB and the release of LPHNs from the microbubbles. The delivery of pCas9/MGMT downregulated MGMT expression, enhanced the sensitivity of glioblastoma cells to temozolomide, and improved the therapeutic efficacy of the drug in glioblastoma treatment. This study demonstrated the potential of combining FUS with microbubbles to significantly enhance the permeability of drugs across the BBB. This approach indicates that ultrasound-responsive CRISPR-Cas9 delivery systems could serve as a non-invasive and safe method for effective gene therapy in CNS diseases and various other conditions [183].

Microbubbles consist of a gas-filled core and a stabilized shell, and this unique structure can amplify the cavitation effect of ultrasound. Upon exposure to ultrasound, the vibration of microbubbles creates temporary pores in the cell membrane, enhancing membrane permeability through a technique known as sonoporation. These characteristics provide significant potential for developing ultrasound-responsive nanofomulations for intracellular delivery of CRISPR-Cas9 systems, which has led to various ongoing studies. For instance, Ryu et al. developed an ultrasound-activated microbubble-conjugated nanoliposome system for Cas9 RNP delivery and applied it to androgenic alopecia treatment. Cas9 RNP targeting the SRD5A2 gene was encapsulated into nanoliposomes approximately 100 nm in size through the film hydration method. These nanoliposomes were then conjugated to SF6 gas-filled microbubbles via disulfide bonds, forming microbubble–nanoliposome complexes (MB-NL). The MB-NL complexes were tested in a testosterone-induced androgenic alopecia mouse model, where the delivered Cas9 RNP recognized and efficiently edited the target gene SRD5A2, restoring hair growth. In particular, external ultrasound stimulation facilitated the penetration of MB-NL into dermal papilla cells (DPCs), while SRD5A2 knockout reduced DPC apoptosis and promoted the proliferation of hair follicle cells. This study demonstrates that CRISPR-based genome editing technology utilizing ultrasound not only holds promise for the treatment of androgenic alopecia but also has the potential to serve as a therapeutic platform for other skin conditions such as skin cancer and melanoma [184].

#### 4.2.3. Magnetic-Responsive CRISPR-Cas9 Delivery

Magnetic-responsive delivery systems integrate magnetic properties into nanostructures, enabling the targeted guidance of materials to specific sites through external magnetic fields. The utilization of external magnetic fields facilitates remote-controlled and targeted drug delivery, minimizes off-target effects, and enables precise gene editing. This approach leverages the unique properties of magnetic fields that interact minimally with the body [185]. Magnetic-responsive systems typically use nanoparticles, such as iron oxide, which are designed to respond to external magnetic stimuli and allow surface functionalization [186]. Moreover, the efficient delivery of non-viral CRISPR-Cas9 systems using magnetic nanoparticles (MNPs) has been investigated.

Magnetic-responsive delivery systems employ magnetofection to perform transfections, resulting in higher editing efficiency. This increased efficiency is attributed to the magnetic properties of the nanoparticles; when placed under a magnetic field, the nanoparticles accelerate sedimentation on the cell surface, increasing the concentration of magnetic complexes [187]. Additionally, nonspecific interactions between the serum components in the culture medium and MNPs were reduced, enhancing cellular uptake without cytotoxicity. This principle suggests that leveraging the magnetic force in genome editing not only facilitates the cellular entry of CRISPR-Cas9-encapsulated delivery systems but also promotes targeted accumulation in specific organs or tissues, providing spatial control for targeted genome editing [188].

In a related study, PEI-MNP complexes with the CRISPR-Cas9 system were used to transfect modified TLR-3 systems expressed in HEK-293 cells via magnetofection. To evaluate HDR and non-homologous end-joining (NHEJ) capabilities, a TLR-3 system incorporating nonfunctional GFP and BFP gene expression cassettes was employed. Depending on the delivery efficiency, CRISPR-Cas9-mediated site-specific DSBs were corrected using HDR (GFP expression) or NHEJ (BFP expression) [113]. The study concluded that PEI-MNPs are promising nanocarriers for CRISPR-Cas9 plasmid delivery.

Kaushik et al. presented the magnetically guided, noninvasive delivery of a nanoplatform containing magnetoelectric nanoparticles (MENPs) conjugated with a Cas9/sgRNA gene-editing system. This MENP-Cas9/gRNA complex was shown to cross the BBB under the influence of a magnetic field, enabling the editing of HIV genes in microglial cells to reduce infection. These MENPs, with a ferromagnetic core and a size of 25 ± 5 nm, were nontoxic up to concentrations of 50 µg and capable of traversing the BBB under static magnetic fields. Concurrently, external alternating magnetic field stimulation induced polarization changes on the MENP surface, breaking the bond between the MENPs and the Cas9/sgRNA complex. As shown in Figure 9e, this mechanism facilitates the release of Cas9/sgRNA within the target cells, enabling effective gene cutting and subsequent genetic alteration [189]. These findings indicate that magnetic-responsive CRISPR-Cas9 delivery systems using MENPs offer precise control of targeted gene editing for CNS disorders and represent a promising gene therapy strategy with in vivo safety and efficacy.

## 5. Perspective: Towards Clinical Application of Advanced CRISPR-Cas9 Delivery Systems

Numerous genetic diseases are caused by mutations in specific base pairs within the DNA, and correcting these mutations is a fundamental goal of gene therapy [190]. The CRISPR-Cas9 technology offers the potential for the insertion, deletion, regulation, and disruption of genes with high precision and efficiency, holding significant promise for treating genetic disorders [191,192,193].

Currently, CRISPR-Cas9 gene therapies are being actively researched, and various clinical studies are ongoing. The FDA has approved the clinical application of CRISPR-based gene therapies for the treatment of sickle cell disease (SCD) [194], β-thalassemia (TDT) [195], transthyretin (TTR) amyloidosis [196], Leber congenital amaurosis type 10 (LCA10) [197], human immunodeficiency virus (HIV) [198], and Duchenne muscular dystrophy (DMD) [199]. These achievements not only demonstrate that the design of targeted delivery systems for efficiently and safely delivering the CRISPR-Cas9 system into the body represents a promising new approach for treating genetic disorders but also suggest that the role of CRISPR-Cas9 in the future gene therapy market will become increasingly significant [200].

Additionally, various delivery systems for targeted delivery are being designed and studied according to the specific characteristics of diseases, [201] and these gene therapies demonstrate excellent loading capacity and high potential for clinical translation.

Nevertheless, CRISPR-Cas9-based gene therapies still face persistent issues, such as the possibility of long-term expression in the body, the efficacy of therapeutic effects, and the induction of immune responses [202]. Among these, the most concerning issue is the safety of CRISPR-based gene-editing technology [203]. Especially the conventional CRISPR-Cas9 system, which induces double-strand breaks (DSBs) and modifies genes through non-homologous end joining (NHEJ) and HDR, faces challenges such as off-target effects and the random nature of HDR and NHEJ [204]. Additionally, during the validation process of CRISPR-Cas9 gene therapies, it has been discovered that large-scale base deletions and chromosomal structural translocations occasionally occur, which carry the potential to cause serious side effects such as malignant tumors. These issues clearly have a critical impact on the safety of gene therapies and act as major factors that significantly hinder their clinical translation potential [205].

To address these issues, besides non-viral delivery systems and stimuli-responsive nanoformulations reviewed before, new technologies such as base and prime editing have been developed, offering enhanced precision and versatility.

A base editor is a tool that can precisely convert specific single bases without inducing DSBs, using catalytically inactive Cas9 (dCas9) for direct gene editing [206]. The major types include the cytosine base editor (CBE), which can convert cytosine (C) to thymine (T) and guanine (G) to adenine (A), and the adenosine base editor (ABE), which enables the conversion of A to G or T to C [207]. However, this technology is limited to base substitutions and is unsuitable for extensive genetic modifications [208].

Prime editing was developed to overcome these limitations, enabling base sequence insertions and modifications without a template such as DSBs, in addition to single-base alterations [209]. Prime editing uses Cas9 nickase and prime editing guide RNA (pegRNA) to create a single-strand break in the DNA and incorporates new sequences through reverse transcription using the primer binding site (PBS) and the reverse transcriptase (RT) template included in the 3’ extension of the pegRNA [210]. The edited sequences were accurately incorporated into the DNA through the cell repair system. This prime-editing technology facilitates a wide range of editing processes, including base substitutions, single-base mutations, short deletions, and insertions [211]. Compared to CRISPR-Cas9 or base editors, prime editors allow for more precise and flexible gene editing. Specifically, they provide more accurate editing than NHEJ-based CRISPR-Cas9 and can induce modifications farther from the original target site than HDR-based systems [212]. Unlike base editors, prime editors can target any base sequence, thereby offering versatility for inducing various types of mutations [213].

Advancements in CRISPR-Cas9 systems, such as improved HDR ratios, optimized target DNA selection, enhanced sgRNA design, and fine-tuned Cas9 activity, have contributed to reduced off-target effects and increased clinical applicability [214]. The development of prime- and base-editing technologies has introduced new possibilities in the field of gene therapy, and these advancements are anticipated to extend to the treatment of a broader range of diseases.

## 6. Conclusions

The CRISPR-Cas9 system has become a core technology of gene therapy, offering the potential to treat genetic disorders and various diseases with remarkable precision. Originally for DNA strand cleavage, CRISPR-Cas9 has now evolved to edit single-base mutations, regulate transcription, and even cleave RNA strands, demonstrating its applicability across multiple domains such as cancer, genetic disorders, and chronic diseases. Still, significant challenges remain regarding widespread clinical application. Off-target effects and the induction of immunogenicity are major factors limiting the therapeutic use of CRISPR-Cas9. The CRISPR-Cas9 system is primarily delivered in the form of plasmids, mRNA, or RNPs; however, it faces obstacles such as rapid clearance by the immune system and difficulties in cellular uptake due to electrostatic repulsion.

Non-viral delivery systems for CRISPR-Cas9, such as LNPs, EVs, polymer-based nanoparticles, AuNPs, and MSNs, are emerging as highly promising alternatives owing to their low immunogenicity and high stability. Nanoparticle-based non-viral delivery technologies enhance biological stability, enable disease-specific targeting, and increase the intracellular delivery efficiency of CRISPR-Cas9 through surface functionalization.

Especially, stimuli-responsive nanoformulations have potential for the spatiotemporal control of CRISPR-Cas9 delivery and activation. Designed to activate only at specific sites in response to internal or external stimuli, these systems enhance the precision of gene editing and minimize off-target effects. Internal stimuli-based systems respond to pH, redox reactions, enzymes, ATP, hypoxia, and RNA, whereas external stimuli-based systems respond to light, ultrasound, and magnetic fields, allowing for adaptation to various therapeutic environments.

Currently, CRISPR-Cas9 gene therapies are being actively researched, and the FDA has approved the clinical application of CRISPR-based gene therapies for the treatment of various genetic disorders. Nevertheless, off-target effects and the low efficiency of HDR remain major factors compromising the safety of gene therapies. To overcome these issues, new technologies such as base editing and prime editing, offering greater precision and flexibility, have been developed. These technologies were designed to address the limitations of conventional CRISPR-Cas9 and represent advancements in genome editing techniques, contributing to the reduction of off-target effects and enhancing clinical applicability.

Despite these advancements, challenges such as improving targeting and cellular uptake efficiency, maintaining stability in vivo, and minimizing immune responses persist, necessitating ongoing research. The fusion of CRISPR-Cas9 technology with nanotechnology is expected to open new paradigms in gene therapy, leading to the development of precise and safe gene therapeutic approaches for clinical applications.

## Data Availability

Not applicable.
